# Post-traumatic Stress Disorder in Women undergoing Caesarean Section: A prospective hospital-based study from Sindh, Pakistan

**DOI:** 10.12669/pjms.41.8.10475

**Published:** 2025-08

**Authors:** Qurat ul Ain Qureshi, Meharunnisa Khaskheli, Roohi Nigar, Feriha Fatima Khidri

**Affiliations:** 1 Qurat ul Ain Qureshi, FCPS Senior Registrar, Department of Obstetrics and Gynecology, Liaquat University of Medical Health Sciences, Jamshoro, Sindh, Pakistan; 2 Meharunnisa Khaskheli, FCPS Chairperson, Department of Obstetrics and Gynecology, Bilawal Medical College, Liaquat University of Medical Health Sciences, Jamshoro, Sindh, Pakistan; 3 Roohi Nigar, FCPS Assistant Professor, Department of Obstetrics and Gynecology, Bilawal Medical College, Liaquat University of Medical Health Sciences, Jamshoro, Sindh, Pakistan; 4 Feriha Fatima Khidri, PhD Associate Professor, Department of Biochemistry, Bilawal Medical College, Liaquat University of Medical Health Sciences, Jamshoro, Sindh, Pakistan

**Keywords:** PTSD, C-section, Elective, Emergency, Pregnancy, Women

## Abstract

**Objectives:**

To study the prevalence of post-traumatic stress disorder (PTSD) in hospital-based population of women undergoing C-section and determine its association with demographic and clinical factors.

**Methods:**

It was a prospective study and conducted between 2022 to 2024 at the Department of Obstetrics and Gynecology, Liaquat University of Medical and Health Sciences (LUMHS), Jamshoro, Sindh, Pakistan. A total of 265 women undergoing C-sections were recruited from multiple hospitals in Sindh, Pakistan, including LUMHS Hyderabad and Jamshoro, Bilawal Medical College, LUMHS, Sindh Government Hospital Qasimabad, and Hashim Medical City Hospital, Hyderabad. PTSD symptoms were assessed using the PTSD Checklist for DSM-5 (PCL-5) at week-1 and followed up at 6 weeks post C-section. The Life Events Checklist 5 (LEC-5) was used to document traumatic experiences. Data was analyzed using the paired samples t-test to compare PTSD scores within groups between week 1 and week 6, and the independent t-test to compare PTSD scores between elective and emergency C-sections. Multivariate logistic regression was performed to identify significant predictors of PTSD while adjusting for confounders. Statistical significance was set at p < 0.05.

**Results:**

The mean age of participants was 29.9 ± 5.3 years, and the mean gestational age was 37.4 ± 1.5 weeks. PTSD scores at one-week post-C-section showed a mean score of 6.72 ± 4.35 for all C-sections, with elective C-sections having a mean score of 6.27 ± 4.35 and emergency C-sections having a mean score of 7.23 ± 4.31 (P-value: 0.077). At six weeks post-C-section, the mean PTSD score was 7.95 ± 6.30 for all C-sections, with elective C-sections having a mean score of 7.34 ± 6.05 and emergency C-sections having a mean score of 8.64 ± 6.53 (P-value: 0.095). A significant increase in PTSD scores from one to six weeks was observed in both groups (p < 0.001). Multivariate logistic regression identified prolonged hospital stay (>5 days) as a significant predictor of PTSD (adjusted odds ratio [AOR] = 42.50, p = 0.013).

**Conclusion:**

PTSD prevalence increased from the immediate postoperative period to six weeks post C-section, highlighting the need for early psychological assessment and intervention in postpartum women, particularly those with prolonged hospital stays following C-sections.

## INTRODUCTION

Child birth is a natural physiological process^1^, and a deeply emotional and challenging experience in a woman’s life. The moment of the joy of welcoming a new child may turn into feelings of fear for newborns and own wellbeing of the mother. This fear of childbirth can arise from anxiety before, during, or after delivery and may be caused by past traumatic experiences, fear of labor pain, concern for the baby, or psychological factors, resulting in severe psychological issues, including post-traumatic stress disorder (PTSD).^2^,^3^ PTSD is a trauma disorder that occurs after direct or indirect exposure to life-threatening events, such as natural disasters, accidents, or violence. Its diagnosis requires assessment of individual for experiencing or witnessing a traumatic event, re-experiencing symptoms, avoidance, negative mood changes, hyperstimulation and irritability, symptoms duration over a month, significant impairment, and exclusion of other causes.^4^,^5^

Caesarean sections (C-sections) are the most common obstetric surgeries and have significantly improved maternal care. However, the risk of maternal death is three times higher compared to normal vaginal delivery (NVD), and it also raises the risk of complications.^6^,^7^ Globally, C-section rates vary widely, from 23.8% to 50% in developed countries and under 10% in developing countries. According to the World Health Organization (WHO), C-sections account for 21% of all births worldwide and could rise to 29% by 2030 exceeding the recommended 15% limit. In Pakistan, C-sections are responsible for 59% of the global maternal death burden, with significant socioeconomic disparities in maternal healthcare.

The C-section rate in urban areas of Pakistan is approximately 25%, highlighting a major public health issue.^6^,^8^,^9^ A study comparing mental health outcomes between natural childbirth and C-sections in Kohat, Khyber Pakhtunkhwa, found that women who underwent C-sections reported more emotional health problems and had a lower mean quality of life score compared to those who had natural childbirth.^10^

Unplanned C-sections can lead to mental health issues like PTSD and postpartum depression. PTSD symptoms include intrusive memories, flashbacks, nightmares, emotional numbness, and avoidance of trauma reminders, along with increased arousal, sleep problems, concentration issues, and irritability. Studies show 24-33% of women who have emergency C-sections experience PTSD symptoms, and 1.5-3% develop PTSD. These symptoms can disrupt bonding with the baby, causing some mothers to feel detached or avoid their child. The link between unplanned C-sections and PTSD is unclear, with varying study results. However, emergency C-sections are generally associated with more distress and fear of future childbirths, impacting overall satisfaction with childbirth.^11^

PTSD following childbirth poses a significant mental health risk for women worldwide.^12^ Nonetheless, it is often overlooked, especially in low-income nations such as Pakistan. Therefore, this study aimed to assess the prevalence of PTSD and its contributing factors in C-section patients in Pakistan and the association between C-section type (elective vs. emergency) and PTSD.

## METHODS

This was a prospective study conducted between years 2022 to 2024 in the Department of Obstetrics and Gynecology, Liaquat University of Medical and Health Sciences (LUMHS), Jamshoro, Sindh, Pakistan.

### Ethical Approval:

The study got approval from the Research Ethics Committee (No. LUMHS/REC/-107; Dated: 17-06-2022).

### Sample Size:

The sample size was calculated using OpenEpi software (https://www.openepi.com/SampleSize/SSPropor.htm), based on a pooled prevalence of PTSD after C-section of 10.7% from a previous study.^13^ At a 95% confidence level, the required sample size was 147. To account for potential nonresponse and ensure sufficient statistical power, the final sample size was increased to 265. The patients were recruited from Gynecology and Obstetrics Units of LUMHS, Hyderabad and Jamshoro (n = 163), Gynecology and Obstetrics Units of Bilwal Medical College, LUMHS (n = 42), Sindh Government Hospital Qasimabad Hyderabad (n = 40) and Hashim Medical City Hospital, Hyderabad (n = 20).

### Participant Recruitment:

Women were recruited for the study during their hospital stay after a C-section using a convenience sample. After obtaining informed consent, gynecologists interviewed the participants and recorded their answers in a questionnaire. Eligible participants were women undergone a C-section (planned or emergency) and were over 18 years of age, provided informed consent and available for follow-up at six weeks postpartum for reassessment of PTSD symptoms. Women with pre-existing psychological disorders, such as depression, anxiety disorders, schizophrenia, or bipolar disorder as diagnosed in their medical records, those taking antipsychotic medication or mood-stabilizing medications (as these could influence PTSD symptomatology), those with a previous history of postpartum mental health problems and were lost to follow up in postpartum period were excluded. The women were followed in a postpartum period and were interviewed for the second time at 6th week of C-section for PTSD scale.

### Study Variables and Data analysis:

The sociodemographic characteristics and other variables were recorded using a predesigned questionnaire. The study included both planned and emergency C-sections, with emergency procedures performed in situations posing immediate threats to the mother or fetus, such as placenta praevia, placental abruption, acute fetal distress, umbilical cord prolapse, or uterine rupture.

The PTSD Checklist 5 (PCL-5) is a 20-item questionnaire, corresponding to the Diagnostic and Statistical Manual of Mental Disorders, Fifth Edition (DSM-5) symptom criteria for PTSD. The self-report rating scale is 0-4 “Not at all,” “A little bit,” Moderately,” “Quite a bit,” and “Extremely” for each symptom. The Life Events Checklist 5 (LEC-5) scale was used to assess the number of traumatic events experienced by the participant, with specific categories for events that happened to them, were witnessed, learned about, part of their job, unsure, or didn’t apply. The data was analyzed using the SPSS, version 24.0. Categorical variables were presented as frequencies and percentages, and comparisons between groups (low PTSD score < 31 vs. high PTSD score ≥ 31) were made using the chi-square test or Fisher’s exact test, where appropriate.

The paired samples t-test was used to compare PTSD scores at week one and week six within the same group and independent t-test to compare scores between elective and emergency C-section. To identify factors associated with PTSD, univariate logistic regression analysis was performed to calculate crude odds ratios (OR) and 95% confidence intervals (CI). Variables with p < 0.2 in univariate analysis were selected for multivariate logistic regression analysis to determine adjusted odds ratios (AOR), adjusting for potential confounders. Statistical significance was set at p < 0.05.

## RESULTS

There were a total 265 women undergoing C-section and meeting the criteria for the study. The mean age of the study participants was 29.9 ± 5.3 years, and the mean gestational age was recorded as 37.4 ± 1.5 weeks. According to the cutoff value of 31 for PTSD scores: At one-week post-C-section, the prevalence of PTSD was 0.8% (2 out of 265). At six weeks post-C-section, the prevalence of PTSD was 3% (8 out of 265) in women undergoing C-section. PTSD scores at one-week post-C-section showed a mean score of 6.72 ± 4.35 for all C-sections, with elective C-sections having a mean score of 6.27 ± 4.35 and emergency C-sections having a mean score of 7.23 ± 4.31 (Table-I).

**Table-I:** PTSD Score at 1-week and 6^th^ week of C-Section.

PTSD Score at 1-week				
	All C-sections	Elective C-Section (Mean ± SD)	Emergency C-Section (Mean ± SD)	P-value (Elective vs Emergency C-Section)
(Mean ± SD)	6.72±4.35	6.27±4.35	7.23±4.31	0.077
	*PTSD Score at 6^th^ week*			
(Mean ± SD)	7.95±6.30	7.34±6.05	8.64±6.53	0.095
*P-value		<0.001	<0.001	

PTSD: Post-traumatic stress disorder; SD: Standard deviation. P-value calculated by independent sample t-test and

* P-value calculated by paired sample t-test at 1^st^ and 6^th^ week.

At six weeks post-C-section, the mean PTSD score was 7.95 ± 6.30 for all C-sections, with elective C-sections having a mean score of 7.34 ± 6.05 and emergency C-sections having a mean score of 8.64 ± 6.53 (P-value: 0.095). The PTSD score range was 2.0-35.0 at one-week and 2.0-40.0 at six weeks. There was no significant difference in mean PTSD scores between elective and emergency C-sections (p = 0.077). However, when comparing PTSD scores within each group over time, a significant increase was observed from the first to the sixth week in both elective and emergency C-sections (p < 0.001). Almost all study participants reported directly experiencing or witnessing events that could be considered potential sources of trauma, as recorded on the LEC-5 scale.

The frequency distribution of variables according to PTSD cutoff score and association of PTSD with demographic and clinical variables in women with PTSD is shown in Tables-II and III. In the unadjusted analysis, the factors that were significantly associated with PTSD included complications during C-section (p = 0.033), referral by general practitioners GPs) or primary healthcare centers (PHCs) or basic health units (BHUs) (p = 0.020), primipara (p = 0.045), fetal gender (p = 0.021). Additionally, prolonged hospital stay (>5 days) was strongly associated with PTSD (p < 0.001). After adjusting for potential confounders, prolonged hospital stay (>5 days) remained a significant predictor of PTSD, with an adjusted odds ratio (AOR) of 42.50 (p = 0.013). Other variables did not retain statistical significance in the adjusted model.

**Table-II:** Frequency distribution and percentage of demographic and clinical characteristics of study participants according to PTSD cutoff score.

Variables		PTSD (n = 265)		P-value
		<31	>31	
Ethnicity	Sindhi	165 (62.3)	4 (1.5)	0.382
	Urdu	14 (5.3)	1 (0.4)	
	Punjabi	15 (5.7)	0 (0)	
	Balochi	17 (6.4)	2 (0.7)	
	Pashto	6 (2.2)	0 (0)	
	Other	40 (15.1)	1 (0.4)	
Education	Illiterate	115 (43.3)	5 (1.9)	0.789
	Primary	59 (22.3)	1 (0.4)	
	Matric/ Intermediate	44 (16.6)	1 (0.4)	
	Graduate/ Postgraduate	39 (14.7)	1 (0.4)	
Husband Education	Illiterate	97 (36.6)	5 (1.9)	0.385
	Primary	41 (15.5)	0 (0)	
	Matric/ Intermediate	55 (20.7)	2 (0.7)	
	Graduate/ Postgraduate	64 (24.2)	1 (0.4)	
Income (Rupees)	<50,000	182 (68.7)	7 (2.6)	0.577
	50, 000-100,000	68 (25.7)	1 (0.4)	
	>100, 000	7 (2.6)	0 (0)	
Relationship status	Married	249 (94)	8 (3.0)	1.000
	Separated/ Widowed	8 (3.0)	0 (0)	
Antenatal visits	0	23 (8.7)	0 (0)	0.388
	1-3	157 (59.2)	4 (1.5)	
	≥4	77 (29.1)	4 (1.5)	
C-section	Elective	134 (50.6)	4 (1.5)	1.000
	Emergency	123 (46.4)	4 (1.5)	
Complications during C-section	Yes	42 (15.9)	4 (1.5)	0.033
	No	215 (81.1)	4 (1.5)	
Type of anesthesia	Epidural/spinal	223 (84.2)	5 (1.9)	0.085
	General	34 (12.8)	3 (1.1)	
Duration of labour	≤ 10 hours	239 (90.2)	6 (2.3)	0.115
	>10 hours	18 (6.8)	2 (0.7)	
Referral status	Self	194 (73.2)	4 (1.5)	0.028
	Midwife/LHV	40 (15.1)	1 (0.4)	
	GP/PHC/BHU	23 (8.7)	3 (1.1)	
Parity	Primipara	30 (11.3)	3 (1.1)	0.064
	Multipara	227 (85.7)	5 (1.9)	
Gestational age	<37 weeks	50 (18.9)	0 (0)	0.359
	≥37 weeks	207 (78.1)	8 (3.0)	
Fetal outcome	Alive	247 (93.2)	7 (2.6)	0.291
	IUD/Stillbirth	10 (3.8)	1 (0.4)	
Number of gestations	Single	253 (95.5)	7 (2.6)	0.143
	Twin	4 (1.5)	1 (0.4)	
Fetal gender	Girl	138 (52.1)	3 (1.1)	0.007
	Boy	117 (44.2)	4 (1.5)	
	Twins (girl, boy)	2 (0.7)	1 (0.4)	
Fetal complications	Yes	22 (8.3)	2 (0.7)	0.157
	No	235 (88.7)	6 (2.3)	
NICU Admission	Yes	40 (15.1)	1 (0.4)	1.000
	No	217 (81.9)	7 (2.6)	
Breast feeding initiated	Yes	188 (71.0)	4 (1.5)	0.222
	No	69 (26.0)	4 (1.5)	
Length of hospital stay	1-5 days	224 (84.5)	2 (0.7)	<0.001
	>5 days	33 (12.5)	6 (2.3)	
Previous mode of delivery	NVD	80 (30.2)	3 (1.1)	0.089
	C-Section	125 (47.2)	1 (0.4)	
	Both	22 (8.3)	1 (0.4)	
	Primipara	30 (11.3)	3 (1.1)	
History of medical disorders	Yes	162 (61.1)	5 (1.9)	1.000
	No	95 (35.9)	3 (1.1)	
Addiction history	Yes	52 (19.6)	0 (0)	0.362
	No	205 (77.4)	8 (3.0)	
Management options	Inpatient	137 (51.7)	5 (1.9)	0.830
	Inpatient and counselling	91 (34.4)	2 (0.7)	
	Inpatient and CBT	29 (10.9)	1 (0.4)	

LHV: Lady Health Visitor; GP: General Practitioner; PHC: Primary Health Care; BHU: Basic Health Unit; IUD: Intrauterine Death; NICU: Neonatal Intensive Care Unit; CBT: Cognitive Behavioral Therapy P-value calculated by chi-square test or Fisher’s exact test.

**Table-III:** Univariate and multivariate logistic regression analysis of factors associated with PTSD following C-Section.

Variables		OR (95% CI)	P-value	AOR (95% CI)	P-value
Ethnicity	Sindhi	1.00 (Ref)	—	1.00 (Ref)	—
	Urdu	2.95 (0.31–28.19)	0.348	1.49 (0.03 – 66.42)	0.836
	Punjabi	0.00	0.999	0.00	0.998
	Balochi	4.85 (0.83–28.47)	0.080	2.43 (0.19 – 31.67)	0.499
	Pashto	0.00	0.999	0.00	0.999
	Other	1.03 (0.11–9.48)	0.978	0.06 (0.00 – 36.25)	0.384
Education	Illiterate	1.00 (Ref)	—		
	Primary	0.39 (0.045–3.414)	0.395		
	Matric/Intermediate	0.52 (0.059–4.601)	0.559		
	Graduate/Postgraduate	0.59 (0.067–5.204)	0.635		
Husband Education	Illiterate	1.00 (Ref)	—		
	Primary	0.705 (0.132–3.758)	0.683		
	Matric/Intermediate	0.303 (0.035–2.655)	0.281		
	Graduate/Postgraduate	0.00	0.998		
Income (Rupees)	<50,000	1.00 (Ref)			
	50, 000-100,000	0.382 (0.046–3.166)	0.373		
	>100, 000	0.000	0.999		
Relationship status	Married	1.00 (Ref)			
	Separated/ Widowed	0.000	0.999		
Antenatal visits	≥4	1.00 (Ref)			
	1-3	0.490 (0.119–2.014)	0.323		
	0	0.000	0.999		
C-section	Elective	1.00 (Ref)			
	Emergency	1.089 (0.267–4.450)	0.905		
Complications during C-section	No	1.00 (Ref)		1.00 (Ref)	
	Yes	5.119 (1.231–21.280)	0.025	1.59 (0.19 – 13.23)	0.667
Type of anesthesia	Epidural/spinal	1.00 (Ref)		1.00 (Ref)	
	General	3.935 (0.899–17.221)	0.069	4.37 (0.32 – 60.57)	0.272
Duration of labour	≤ 10 hours	1.00 (Ref)		1.00 (Ref)	
	>10 hours	4.426 (0.833–23.522)	0.081	6.92 (0.03 – 1899.82)	0.499
Referral status	Self	1.00 (Ref)		1.00 (Ref)	
	Midwife/LHV	1.212 (0.132–11.137)	0.865	0.10 (0.00 – 50.76)	0.465
	GP/PHC/BHU	6.326 (1.332–30.048)	0.020	2.85 (0.19 – 42.01)	0.445
Parity	Multipara	1.00 (Ref)		1.00 (Ref)	
	Primipara	4.540 (1.032–19.967)	0.045	0.885 (0.001– 641.338)	0.971
Gestational age	≥37 weeks	1.00 (Ref)			
	<37 weeks	0.00	0.997		
Fetal outcome	Alive	1.00 (Ref)			
	IUD/Stillbirth	3.529 (0.395 - 31.484)	0.259		
No. of gestation	Single	1.00 (Ref)		1.00 (Ref)	
	Twin	9.036 (0.891 - 91.616)	0.063	0.00	
Fetal gender	Girl	1.00 (Ref)		1.00 (Ref)	
	Boy	1.573 (0.345 - 7.170)	0.559	3.16 (0.26 – 37.87)	0.363
	Twins (girl, boy)	23.0 (1.61 - 328.50)	0.021	1.08×10¹□ (0.00)	0.999
Fetal complications	No	1.00 (Ref)			
	Yes	3.561 (0.678 - 18.706)	0.134	2.10 (0.11 – 41.55)	0.627
NICU Admission	No	1.00 (Ref)			
	Yes	0.775 (0.093 - 6.471)	0.814		
Breast feeding initiated	Yes	1.00 (Ref)			
	No	2.725 (0.663 - 11.195)	0.164	2.98 (0.09 – 97.94)	0.540
Length of hospital stay	1-5 days	1.00 (Ref)			
	>5 days	20.364 (3.944 - 105.136)	<0.001	42.50 (2.17 – 831.88)	0.013
Previous mode of delivery	C-Section	1.00 (Ref)			
	NVD	4.687 (0.479 - 45.851)	0.184	9.44 (0.23 – 394.92)	0.239
	Both	5.682 (0.343 - 94.243)	0.225	5.74 (0.007 – 4602.93)	0.608
	Primipara	12.500 (1.256 - 124.426)	0.031	2.51 (0.14 – 44.13)	0.529
History of medical disorders	No	1.00 (Ref)			
	Yes	0.977 (0.228 - 4.182)	0.975		
Addiction history	No	1.00 (Ref)			
	Yes	0.00	0.997		
Management options	Inpatient	1.00 (Ref)			
	Inpatient and counselling	0.602 (0.114 – 3.171)	0.550		
	Inpatient and CBT	0.945 (0.106 – 8.392)0.959	0.959		

OR: Odds Ratio; AOR: Adjusted Odds Ratio; CI: Confidence Interval; Ref: Reference Category; C-Section: Cesarean Section; NICU: Neonatal Intensive Care Unit; NVD: Normal Vaginal Delivery; LHV: Lady Health Visitor; GP: General Practitioner; PHC: Primary Health Care; BHU: Basic Health Unit; IUD: Intrauterine Death; CBT: Cognitive Behavioral Therapy.

## DISCUSSION

This study investigates the prevalence and risk factors associated with PTSD in women undergoing C-section. The findings provide valuable insights into the demographic and clinical characteristics influencing postpartum psychological outcomes following C-sections and adds evidence from a lower-middle-income country like Pakistan, where postpartum psychological disorders are often underdiagnosed and untreated. With a PTSD score cutoff of 31, prevalence was 0.8% at one-week and 3% at six weeks post-C-section. Mean scores increased from 6.72 ± 4.35 to 7.95 ± 6.30 over time significantly, with no significant difference between elective and emergency C-sections. Scores ranged from 2.0 to 40.0, indicating symptom variability. Nearly all participants reported direct exposure to potentially traumatic events based on the LEC-5 scale, highlighting the widespread nature of trauma experiences. Our study highlights that PTSD prevalence post-C-section may be lower than expected, especially in women without psychiatric comorbidities or major obstetric complications. The multicenter design enhances generalizability, and inclusion of both elective and emergency C-sections ensures broader applicability. Using DSM-5 criteria and validated tools (LEC-5) strengthens reliability, while adjusting for confounders provides a clearer association between C-sections and PTSD.

In consistent to our study findings, Grisbook et al^14^, found no direct association between C-section type and PTSD symptoms. Women who experienced an emergency C-section had a 0.24 increase in PTSD scores (0.6±1.5) compared with women undergoing elective C-section. Mahmoodi et al.^15^ found a 6.2% PTSD prevalence among Iranian women, with higher rates following emergency C-sections (8.4%) than elective ones (4.5%), though differences were not statistically significant. Poor social support was a key risk factor in their study. Dekel et al.^16^ found that women who underwent unplanned C-sections had the highest risk of childbirth-related PTSD (41.2%), followed by assisted vaginal (24.5%) and planned cesarean (17.4%) deliveries. In contrast, PTSD rates were lower among those with natural (12.5%) and vaginal (14.7%) births.

We found a low prevalence of PTSD post C-section in our study compared to other studies.^13^ There may be various reasons for that such as majority of the participants were multiparous and had previously experienced C-section uneventful, most of the women had elective C-section and did not suffer any complications in present C-Section. Apart from that included women had no previous psychiatric comorbidities. It has been reported that approximately 40% of women described their C-section experience as traumatic, with a notable disparity between emergency C-Section (71.4%) and elective C-section (19.6%).^14^ Schwab et al.^17^ reported that 21.15% of multiparous women with EMCS met the full PTSD diagnostic criteria at six weeks postpartum. This prevalence included both new and pre-existing PTSD cases. After excluding cases with prior trauma-related PTSD or subsyndromal forms, the incidence rate of postpartum PTSD was below 8% at six weeks. Similarly, the low PTSD prevalence in our study may be due to the exclusion of women with a history of mental illness or previous postpartum mental health problems.

Modaress et al.^18^ found significantly higher PTSD rates in EMCS (43.8%) compared to ELCS (23.2%) and vaginal deliveries. Conversely, the study by Lopez et al.^3^ did not find a significant association between delivery mode and PTSD outcomes. Additionally, van Heumen et al.^19^ suggested that psychosocial factors might outweigh delivery mode in predicting postpartum mental health outcomes.

In this study, 3% of participants met DSM-5 criteria for PTSD after C-Section, with significantly higher rates observed at six weeks compared to one-week post C-section. This disparity underscores the traumatic nature of emergency surgical interventions, potentially exacerbated in later weeks compared to initial week. A notable finding was the association between prolong hospital stay >5 days and PTSD in our study. Similarly, Schaal et al.^20^ reported that elevated postoperative anxiety levels in patients with extended hospitalization after C-sections. Previous studies have linked adverse fetal outcomes with increased risk of postpartum psychological disorders due to associated maternal distress.^21^ Although breastfeeding initiation emerged as a protective factor, potentially mitigating maternal trauma post C-section in previous studies. PTSD after C-Section was less prevalent among mothers who initiated breastfeeding, aligning with research suggesting that breastfeeding supports maternal infant bonding and reduces stress through elevated prolactin levels. Conversely, the absence or early cessation of breastfeeding was associated with heightened PTSD risk.^22^ We did not find any significant association of other demographic and clinical factors which might be due to less prevalence of PTSD in our cohort.

### Limitations

This study has certain limitations. PTSD was assessed at one and six weeks postpartum, and longer-term psychological effects beyond this period were not evaluated. Selection bias cannot be ruled out, as participation was voluntary and might have excluded women with more severe psychological distress. Furthermore, the use of convenience sampling may limit the generalizability of the findings to the broader population. However, to minimize selection bias, we recruited participants from multiple centers and included both elective and emergency C-sections to ensure a diverse sample. Future studies should incorporate randomized recruitment strategies to further improve the validity and generalizability of results.

## CONCLUSION

PTSD prevalence increased from the immediate postoperative period to six weeks post C-section, highlighting the need for early psychological assessment and intervention in postpartum women, particularly those with prolonged hospital stays following C-sections. Healthcare providers should prioritize identifying and supporting women at heightened risk, particularly those undergoing emergency procedures or experiencing complications during pregnancy. Future research should further explore regional variations in PTSD prevalence and the effectiveness of tailored interventions in diverse healthcare contexts.

### Authors’ Contribution:

**QAQ:** Conception, design, drafting, data collection, responsible for integrity of manuscript.

**MK:** Supervision, critical review of manuscript, revision and approval of final version.

**RN:** Methodology, data collection, and data interpretation. Critical Review

**FFK:** Drafting, design, data analysis and interpretation. Critical analysis

All authors have read and approved the final version.
